# Assessing the acceptability of dried blood spot testing for HIV and STBBI among Métis people in a community driven pilot project in Alberta, Canada

**DOI:** 10.1186/s12913-022-08763-z

**Published:** 2022-12-08

**Authors:** Rachel Landy, Danielle Atkinson, Kandace Ogilvie, Raye St. Denys, Carrielynn Lund, Catherine Worthington, on behalf of the DRUM & SASH team

**Affiliations:** 1 School of Public Health and Social Policy, HSD Building, Room B202, University of Victoria, PO Box 1700 STN CSC, Victoria, BC V8W 2Y2 Canada; 2 Shining Mountains Living Community Services, 4925 46 St, Red Deer, AB T4N 1N2 Canada; 3 Communities, Alliances and Networks, PO Box 2978, Fort Qu’Appelle, SK S0G1S0 Canada

**Keywords:** Dried blood spot testing, Métis health, HIV, HCV, HBV, Syphilis, Métis evaluation, STBBI, Indigenous health

## Abstract

**Background:**

Little literature exists on culturally grounded approaches for addressing human immunodeficiency virus (HIV) and sexually transmitted and blood-borne infections (STBBI) among Métis people. The goal of this mixed-methods research was to explore the experiences of Métis community members participating in a dried blood spot testing (DBST) for HIV/STBBI pilot for Métis communities in Alberta, Canada, with the aim of assessing the acceptability of this testing method.

**Methods:**

Grounded in community-based and Indigenous research approaches and working in partnership with a Métis community-based organization, data collection included a survey and four gathering circles with Métis DBST recipients at one of two community events, and semi-structured interviews with three DBST providers.

**Results:**

Twenty-six of the 30 DBST recipients completed surveys, and 19 DBST recipients participated in gathering circles. Survey results suggest DBST is a highly acceptable STBBI testing method to Métis community members. Thematic analysis of gathering circle and interview transcripts revealed four broad themes related to the participants’ experiences with DBST related to its acceptability (i. ease of DBST process, ii. overcoming logistical challenges associated with existing STBBI testing, iii. Reducing stigma through health role models and event-based, and iv. Métis-specific services).

**Conclusions:**

These findings illustrate the potential for DBST to be part of a culturally grounded, Métis-specific response to HIV and STBBI.

**Supplementary Information:**

The online version contains supplementary material available at 10.1186/s12913-022-08763-z.

## Background

The Métis, one of the three legally recognized groups of Indigenous Peoples in Canada, account for approximately 33% of the country’s Indigenous population [1]. The Métis are distinct Indigenous Peoples that arose from the marriage of European settlers (typically fur traders) with First Nations women, as early as the seventeenth century [2]. Their offspring, who were half Indigenous and half European, were often excluded from their First Nation and European settler communities, and became reliant on themselves, forming their own communities. Subsequently, these communities developed unique cultures and traditions which formed the beginning of what we now know as the Métis Nation [3].

Although distinct from other Indigenous Peoples, the Métis experience health disparities, similar to other Indigenous Peoples in Canada, resulting from the ongoing impacts of colonization, which include culturally unsafe and inequitable health care services, as well as oppressive policies [4]. Health disparities experienced by Indigenous Peoples in Canada, including the Métis, include overrepresentation in rates of Human Immunodeficiency Virus (HIV), Hepatitis C (HCV), and other sexually transmitted and blood borne infections (STBBI) [5]. For instance, although Indigenous Peoples account for 4.9% of Canada’s population, in 2017, 20.1% of HIV-positive tests with a race/ethnicity identifier were among Indigenous individuals (First Nation (17.4%); Métis (2.3%); Inuit (0.2%); and Indigenous unspecified (0.3%)); however, only 49.3% of reported HIV cases had an attached ethnic identifier nationwide [1, 6]. Similarly, the rate of Hepatitis C (HCV) infection is estimated to be approximately 5 times the rate in Indigenous populations than non-Indigenous populations [7, 8]. Additionally, Indigenous Peoples in Canada are disproportionately affected by other STBBIs [5, 9]. Across Canada, rates of other STBBI, such as syphilis, have increased significantly since 2010, and the overrepresentation of Indigenous Peoples in the STBBI statistics is a trend that is mirrored among Indigenous communities worldwide [10–13]. However, in Canada, the lack of consistency regarding ethnic identifiers, reporting standards, and surveillance data across the provinces and territories make reliable statistics for STBBI challenging and reliable Métis-specific data is unavailable.

Diagnosis remains a barrier to prevention and treatment of HIV and other STBBI among Indigenous Peoples, as well as the general population. Of the approximately 62,050 individuals estimated to be living with HIV in Canada, 12.5% of positive individuals have not been diagnosed [14]. Even more notable, of the approximately 220,697 to 245,987 individuals living with HCV in Canada, it is estimated that 44% are undiagnosed [7, 8]. Considering the overrepresentation of Indigenous Peoples in the STBBI data and the estimation that many people remain undiagnosed, it is imperative that Indigenous Peoples have access to acceptable and culturally safe STBBI testing services. Yet, it is well documented that globally many Indigenous Peoples, including the Métis, lack access to culturally safe health services (i.e., services provided by culturally competent health care providers in a context that acknowledges the impact of colonization and recognizes the potential for power imbalances and institutional discrimination), which can perpetuate health disparities [5, 11–13, 15–20].

Dried blood spot testing (DBST) is a novel approach to STBBI testing in Canada which may improve accessibility to culturally safe STBBI testing within Métis communities. It is a portable method of testing blood for the presence of antibodies, and involves using a lancet to prick the fingertip, which is blotted onto a filter paper, then dried and shipped to a laboratory for testing [21]. DBST can be administered by individuals who are not health care providers/clinicians and has been piloted within First Nation communities with limited health care infrastructure [22]. DBST is an alternative to phlebotomy, which is the current standard practice for testing for HIV, HCV, syphilis, and other STBBI across Canada.

The acceptability of a health care intervention to service users and providers is critical for successful implementation [23]. Acceptability is a “multi-faceted construct that reflects the extent to which people delivering or receiving a health care intervention consider it to be appropriate, based on anticipated or experiential or cognitive emotional responses to the intervention” ([23] , p. 8). Within Indigenous health services contexts, cultural appropriateness and cultural safety are important considerations related to acceptability of an intervention [24]. Hence, the objective of this study was to explore Métis community members’ experiences with DBST with the aim exploring the acceptability of DBST as a method for testing for STBBI, including factors related to increasing accessibility to STBBI testing within a Métis cultural context.

## Methods

### Project partnership

This study developed from a collaboration between the CIHR-funded DRUM & SASH Implementation Science team that aims to support the development and implementation of community and culturally led interventions to address HIV, HCV and other STBBI and related mental health issues and Shining Mountains Living Community Services (Shining Mountains), on behalf of the Métis Nation of Alberta (MNA). Shining Mountains is an Indigenous-run agency located in Central Alberta offering a wide variety of health and social services to Indigenous Peoples across Alberta, Canada and the DRUM & SASH team is comprised of First Nation, Métis, and non-Indigenous community-based researchers, clinicians, health providers, Elders, and knowledge users. The Métis Nation of Alberta and Shining Mountains expressed interest in offering STBBI testing at community events to increase awareness and reduce stigma regarding HIV and STBBI in the Métis community. Facilitated by their partnership with DRUM & SASH, they partnered with the Public Health Agency of Canada’s National Laboratory for HIV Reference Services (NLHRS), Alberta Health Services (AHS), and the Red Deer Sexual Health Clinic to pilot DBS testing for STBBI within Métis community contexts.

### Pilot events

For this pilot, DBST was used to test for HIV, HCV, HBV, and syphilis antibodies and offered at two Métis community events: the DBST launch event (September 2019) and the annual Métis health forum hosted by the MNA, which features a health fair with several dozen booths distributing information on training programs, resources, and services geared towards Métis people (November 2019). Three members of staff from Shining Mountains were trained to give dried blood spot tests at the pilot events.

### Research approach

Using community-based and Indigenous research approaches prioritizing Métis perspectives, this concurrent triangulation mixed-method study explored the perspectives and experiences of Métis service providers and DBST recipients during the DBST pilot events to describe its perceived acceptability [25]. Four of the co-authors (DA, RS, CL, & KO) identify as Métis women and were involved in the development and implementation of this pilot project in varying capacities. This study was conducted by DA as part of the requirements for a Master of Public Health degree. RL and CW were members of DA’s supervisory committee and supported the development and implementation of the research, analysis of the data, and presentation of the research findings, along with CL, KO, and RS who informed this study at all stages from their perspectives as leaders within the Métis community.

To explore the acceptability of DBST from the perspective of Métis test recipients participating in the DBST pilot, all Métis community members who received DBST at one of the two pilot events were offered an information letter about the study and invited by DA to complete a brief, self-administered pencil-and-paper survey in-person immediately after their test and participate in a gathering circle the same day and in a private area in the same venue as the DBST. A gathering circle is similar to a focus group or sharing circle, which is a commonly used data collection method in First Nations research [26, 27]; however, a gathering circle incorporates Métis culture and practices. The term “gathering circle” was coined by Métis co-authors, RS and CL, to describe this Métis-specific data collection method. Survey questions addressed participant demographics including year of birth, ancestry, spoken languages, place of residence, sex, gender and sexual identities, previous testing experience, and acceptability of DBST through questions related to participants perceptions of and attitudes toward the DBST testing process, including factors related to the ease of testing, willingness to use this service again, and willingness to recommend this testing method to others [28]. The survey tool development was led by DA with guidance from other team members, including Métis knowledge users, who provided feedback on wording and constructs being assessed. To complement the survey data, Métis DBST recipients were also invited by DA to participate in a gathering circle to further explore their experiences with DBST at the pilot events to better understand this intervention’s acceptability and how DBST may increase accessibility to STBBI for Métis communities. Gathering circles, lasting approximately 45 miuntes each, were hosted by DA at each pilot event [25, 29, 30]. Participants were provided with a $25 gift card as a token of appreciation.

To explore the acceptability of this intervention from the perspective of Métis service providers, all Shining Mountains employees trained to administer DBST at the pilot events were invited to participate in a telephone interview lasting approximately 45 minutes after the pilot events. DBST providers were offered a $25 gift card as a token of appreciation. Ethics approval was obtained from the University of Victoria’s Human Research Ethics board (certificate # 18–1179); participants gave written informed consent prior to participating in the study.

### Data analysis

Survey data were analysed using descriptive statistics in Microsoft Excel [31]. Interviews and gathering circles were conducted by DA, audio-recorded, transcribed in full, and managed in NVivo 12 software [32]. Transcripts were read for familiarity, analysed for themes derived from the data using a holistic content analysis approach, and then the themes were organized into meaningful text [29, 30, 33]. Data were first coded by DA, and then colleague review was provided by RL and CW. The Consolidated Criteria for Reporting Qualitative Research (COREQ) checklist was used to guide the reporting of this study [34].

## Results

Thirty people received DBST during the pilot. Of those, 26 who self-identified as Métis opted to participate in this research study and completed the survey and nineteen accepted the invitation to participate in a gathering circle. Participation in the study was voluntary, and those who received DBST but declined the opportunity to participate in the study were not questioned on this decision. Three Métis service providers were interviewed individually about their experiences receiving training and administering DBST during the event. The majority (76%) of gathering circle participants were self-identified Métis women; all three DBST provider interviewees identified as Métis women.

### Survey results

Survey results, including participant characteristics, previous STBBI testing experiences, and perceived acceptability of DBST are presented in Tables 1, 2, and 3, respectively.

**Table 1:** Participant Characteristics

Characteristic	Total (***n*** = 26)	
	*n*	%
Sex		
Female	19	73%
Male	7	27%
Year of Birth (*n* = 24)^a^		
Mean-reported	1965	
Range-reported	1943–1997	
Place of residence (*n* = 25)^b^		
City	20	77%
Rural/isolated/Métis settlement	5	19%
Languages spoken		
English	26	100%
Cree or Michif	4	15%
French	4	15%

^a^Two participants did not indicate a birth year

^b^One participant did not indicate a place of residence

**Table 2:** Previous STBBI Testing Experiences

Question	Total (***n*** = 26)	
Have you ever been tested for HIV?	**n**	**%**
Yes	11	42%
No	13	50%
Unsure	2	8%
If so when was your last HIV test?^a^ (*n* = 11)	**n**	**%**
In the past 3 months	1	4%
4–6 months ago	–	–
7–12 months ago	1	4%
Longer than a year ago	9	35%
Have you ever been tested for HCV?	**n**	**%**
Yes	9	35%
No	12	46%
Unsure	5	19%
If yes, when was your last HCV test? (*n* = 9)	**n**	**%**
In the past 3 months	1	4%
4–6 months ago	–	–
7–12 months ago	–	–
Longer than a year ago	8	31%
Have you ever been tested for HBV?	**n**	**%**
Yes	5	19%
No	14	54%
Unsure	7	27%
If yes, when was your last HBV test? (*n* = 5)	**n**	**%**
In the past 3 months	1	4%
4–6 months ago	–	–
7–12 months ago	–	–
Longer than a year ago	4	15%
Have you ever been tested for syphilis?	**n**	**%**
Yes	4	15%
No	18	69%
Unsure	4	19%
If yes, when was your last syphilis test? (*n* = 4)	**n**	**%**
In the past 3 months	1	4%
4–6 months ago	–	–
7–12 months ago	–	–
Longer than a year ago	3	12%

**Table 3:** Perceived Acceptability of DBST

Question	Total (***n*** = 26)	
I would recommend this testing method to friends or family	**n**	**%**
Strongly Agree	20	77%
Agree	6	23%
Neither Agree nor Disagree	–	–
Disagree	–	–
Strongly Disagree	–	–
I thought this type of testing was easy	**n**	**%**
Strongly Agree	22	85%
Agree	3	12%
Neither Agree nor Disagree	1	4%
Disagree	–	–
Strongly Disagree	–	–
I would use this type of testing again	**n**	**%**
Strongly Agree	23	88%
Agree	2	8%
Neither Agree nor Disagree	1	4%
Disagree	–	–
Strongly Disagree	–	–
I received enough information today about HIV/HCV/HBV/ syphilis	**n**	**%**
Strongly Agree	20	77%
Agree	5	19%
Neither Agree nor Disagree	–	4%
Disagree	–	–
Strongly Disagree	–	–
I will encourage family and friends to get tested	**n**	**%**
Strongly Agree	20	77%
Agree	2	8%
Neither Agree nor Disagree	4	15%
Disagree	–	–
Strongly Disagree	–	–
Overall, my testing experience was positive	**n**	**%**
Strongly Agree	20	77%
Agree	5	19%
Neither Agree nor Disagree	1	4%
Disagree	–	–
Strongly Disagree	–	–

#### Participant characteristics

Twenty-six (26) self-identified Métis individuals completed the survey (see Table 1). Most (19, 73%) identified as female, and participant-reported mean birth year was 1965 (range: 1943–1997, mean age 54 years) (2 participants did not indicate a birth year). The majority (77%) of survey participants lived in an urban environment. Four (15%) participants indicated they spoke an Indigenous language. Of note, results regarding information about sexual identity and gender are not reported as more than half the participants did not indicate a response.

#### Previous testing experience

Thirteen of 26 participants (50%) indicated they had never been tested for HIV before their DBST (see Table 2). Of those who had previously tested for HIV, nine respondents (35% of all respondents) indicated their last test occurred over a year ago. Twelve respondents (46%) indicated they had never been tested for HCV previously and eight respondents (31%) indicated they had been tested for HCV more than a year ago. Fourteen out of 26 participants (54%) indicated they had never been previously tested for HBV. Four participants (15%) indicated they had previously been tested for syphilis, and four (15%) were unsure if they had previously been tested. Of the four that indicated they had previously been tested for syphilis, three indicated their test was over a year ago, and one was in the last 3 months.

#### Acceptability of DBST

Survey participants were asked to indicate their level of agreement with statements about DBST on Likert scales (see Table 3). Overall, the responses indicated that the participants perceived DBST favourably, suggesting that they found this testing experience acceptable. All 26 survey participants indicated that they strongly agreed or agreed with the statement “I would recommend this testing method to friends or family”, and 25 (96%) indicated they strongly agreed or agreed that DBST was “easy”; while 24 (92%) indicated that they strongly agreed or agreed that they would receive DBST again. In addition, 25 (96%) indicated they strongly agreed or agreed that they received enough information about HIV, HCV, HBV and syphilis during their testing experience.

### Gathering circles and interview results

Thematic analysis of gathering circles and interview transcripts revealed four themes related to increasing accessibility of STBBI testing informing the acceptability of DBST as a STBBI testing strategy within a Métis community context. These themes include: i. ease of the DBST process, ii. overcoming barriers associated with phlebotomy, iii. Reducing stigma – role modelling health behaviours in the context of event-based testing, & iv. Métis specific services. These themes are discussed below.

#### Ease of DBST Process

Many participants described their DBST experience positively, using words including “easy”, “fast”, “quick”, “simple”, and “comfortable”, and indicated there was minimal pain or discomfort when receiving DBST. One participant shared their surprise at how easy the process was, “Well they can test for 4 or 5 different diseases all at once within 60 seconds, man, and no pain. None. Done. Wow.” (DBST recipient). Similarly, another participant stated, “Easy. That’s the key word right there, it’s so easy, you just go in and you go out. Done.” (DBST recipient)

#### Overcoming barriers associated with phlebotomy (standard practice)

Gathering circle participants felt DBST could reduce barriers to STBBI testing associated with existing testing services (i.e., phlebotomy) for Métis people. The challenges associated with phlebotomy included: distance to service providers, length of time required for appointments, follow-up visits with multiple providers (i.e., receive a requisition from one provider and follow-up with another provider for a blood draw). These barriers were described as amplified for those in more rural settings and smaller communities since receiving care often requires time away from work and access to transportation which can also increase financial burdens:

*You know, like, right now you have to go to the lab, drive, like us rural people, we have to drive to [community A] or [community B] go to the lab, wait there for an hour, get your test, go home, then you have to drive back, then oh you have to make an appointment with your doctor to get your results read … like, you know, I have to drive like 40 miles every 3 months to meet those two appointments/trips, you know, and it’s costly.* (DBST recipient)

Similarly, when participants were asked what they thought friends and family might think of DBST, one person indicated that their friends or family were not likely to get tested outside of something like DBST offered at an event “because they have to go to a lab to get it done” (DBST recipient). Another participant agreed and stated that they thought DBST would have to come to their community (a Métis settlement) in order to get family, friends, or other community members tested for HIV or STBBI.

#### Reducing Stigma – Role modelling health behaviours in the context of event-based testing

##### Stigma related to HIV/STBBI & testing

Many participants felt there was a general lack of knowledge, information, and awareness about HIV/STBBI within Métis communities. Some described these knowledge gaps as contributing to stigma about HIV/STBBI within the Métis community, including stigma related to testing and treatment. For instance, one participant stated,

*There’s a lot of people that I know [who] are still in the stigma of, “It’s a death sentence”, you know. Even I find myself looking down at these people, and I shouldn’t because I know it’s not.* (DBST recipient)

Although some participants noted that stigma related to STBBI testing, sexual health, and sex as topics of conversation has improved over time, they also felt stigma continues to be a pervasive issue within Métis families and communities. For instance, one participant indicated that the lack of openness about sexual health, HIV, and STBBI resulting from stigma contributes to fear, saying, “Some people are very scared just of the idea of talking about AIDS or HIV or syphilis,” which subsequently leads people to refrain from STBBI testing (DBST recipient). Thus, DBST recipients and providers identified stigma as a barrier to STBBI testing.

While participants described stigma as a barrier to STBBI testing, they also expressed that DBST may help to reduce stigma as it allows for testing to be done in a community event setting where testing behaviours can be role modeled.

##### Role modelling health behaviours in the context of event-based testing

Participants in the gathering circles identified that hosting DBST at Métis community events allowed for health promoting behaviors to be modeled by and for community members, which they saw as a strategy for reducing stigma associated with STBBI testing. Additionally, they identified that having DSBT offered at a Métis wellness gathering in an event-based format with other health-related information and activity booths helped to normalize STBBI testing by making it visible. Some participants felt that normalizing and increasing the visibility of STBBI testing may also contribute to reducing stigma associated with being tested and may encourage others to be tested.

DBST recipient 1: *I can see it in like gatherings like this where we’re all together, we’re not being singled out, like we’re all Métis Peoples, we’re all doing the testing, we’re looking after our health.*

DBST recipient 2: *And that’s it. We’re being role models in the sense of yes, I’ve taken the time to look after my health or to see if there’s something more to take care of.*

Thus, DBST recipients felt that providing DBST testing in a community event format where community members could model health promoting behaviours may contribute to normalizing STBBI testing and reduce stigma related to STBBI testing which may ultimately improve STBBI testing uptake within their communities.

#### Métis-specific services

DBST recipients and providers commented on the lack of Métis-specific services in general, and increased comfort with STBBI testing at the DBST pilot due to the Métis context.

Some DBST recipients described a lack of Métis-specific STBBI services and indicated that DBST could address this gap; this sentiment was also described by one of the testing providers. In her interview, she spoke about the need for Métis-specific services regarding HIV, STBBI and sexual health in general:

*I would love to be able to see more being developed. Because obviously there’s a need out there for Métis Peoples to have Métis-specific services. I think that even with just the few dried blood spot events that we’ve had it shows that Métis Peoples are willing to try something like that when it is being offered by their own community. And I think that you know especially in the bigger [city] centres Métis stuff would be fantastic. I think a lot of people if they know about it, they would be more than willing to access it.* (DBST provider)

This provider felt that the there is a need and desire for Métis-specific services and that Métis-specific services may improve access to services for Métis people.

DBST recipients emphasized that having Métis DBST providers within a Métis community event context contributed to their comfort receiving STBBI testing during this pilot. DBST providers were described by test recipients as personable and likeable, helping to increase the comfort of those being tested. One participant stated:

*[DBST provider] talked and it was nice and it was actually more relaxing than doing the diabetic test … this was like the same type, like getting the same puncture, but it was relaxing and I wasn’t used to that because it was just, you know, natural, more natural, there was no tension around it.* (DBST recipient)

This comfort was similarly experienced by the DBST service providers. For instance, one provider, when asked about the potential cultural safety of DBST in this pilot (a community-driven Métis specific service), stated, “I think they [DBST recipients] feel, or you know, the ones that I chatted to about it felt more comfortable, especially knowing that it was a Métis person doing their testing”.

In addition to the comfort created by having Métis service providers administering DBST at a Métis event, all three DBST providers suggested that this process could allow for Métis DBST recipients to be connected to Métis-specific services in the instance of a positive test result, such as connecting individuals with a Métis Elder in a safe environment, and indicated this could lead to improved cultural safety of STBBI services for Métis people. Overall, many participants indicated a preference for Métis-specific services and commented on the lack of Métis-specific health and social services. DBST allowed for Métis-specific service delivery as it was offered at Métis-specific events, and Métis community members were trained to provide it which may increase access to culturally safe STBBI testing for Métis community members.

## Discussion

### Acceptability of DBST

To our knowledge, this is the first study of the experiences of Métis people participating in DBST with the aim of better understanding the acceptability of DBST within a Métis community context. Survey participants rated their experiences with DBST during the pilot highly, indicating that they found it to be an acceptable method for STBBI testing: all of those tested would recommend DBST to friends or family, 96% indicated the testing was “easy” and 92% of participants were willing to use DBST again. Thematic analysis of gathering circle and interview transcripts provided greater insight into the participants’ experiences of DBST and contributed to a deeper understanding of its acceptability within a Métis cultural context. Ultimately, these themes relate to the acceptability of DBST for STBBI testing as a strategy that may improve access and reduce barriers to testing for Métis communities.

Survey results indicated that half (50%) of the participants had never received an HIV test prior to their DBST, nearly half (46%) had not received an HCV test prior to this pilot event, and more than half (54%) of the participants had never had an HBV test and very few participants had ever been tested for syphilis before this event suggesting that the “ease” of DBST may have the potential to increase access to STBBI testing for Métis community members. Likewise, several of the themes emerging from the qualitative analysis of the interview and gathering circles with test recipients and providers provide greater context to the participants’ views regarding the acceptability of DBST within this context and suggest that DBST may improve access to STBBI testing for Métis communities. Both providers and recipients described the DBST process as easy: it was easy to get tested and it was easy to have community members be trained to administer DBST. Our findings regarding the ease and simplicity of the DBST process are corroborated by existing research, which has shown that DBST is straightforward enough for individuals to provide self-collected samples and may lead to increase in STBBI testing [22, 35]. Additionally, portability was seen as a beneficial quality, contributing to the “ease” of DBST, by both DBST providers and recipients, and was identified by participants as a strategy for increasing STBBI testing rates and accessibility for Métis community members in rural/remote areas. The portability of DBST has also been recognized as a factor that may increase testing rates among other populations, including First Nations communities, incarcerated individuals, individuals who inject drugs, and may also be useful in resource-limited settings where phlebotomy may be unsuitable [22, 36–38].

The potential for DBST to overcome challenges associated with existing testing processes for Métis community members were discussed at length by participants. For some, these challenges represent barriers which can prevent community members, including themselves, from engaging in the STBBI testing. Currently, there is a lack of research exploring barriers to STBBI testing for Métis communities; however, similar barriers to STBBI testing have been identified within a variety of populations, including in Indigenous populations, in Canada, Australia, Brazil, and the United Kingdom [11, 12, 39]. These barriers include: internalized stigma, negative perceptions of HIV, not being recommended by a physician or healthcare practitioner to get tested, poor quality of testing services, lack of cultural safety of testing services, a poor understanding of HIV, geographic distance, the need to return for results, and a lack of time [11, 12, 39]. Our findings suggest that participants felt DBST was a recommendable testing strategy as it may increase access to STBBI testing for Métis community members by overcoming many of these barriers to testing associated with standard testing practices (i.e., phlebotomy).

### Reducing stigma and increasing awareness

Reducing stigma related to HIV/STBBI testing and increasing awareness about HIV/STBBI testing services may improve access to testing for Métis people. Our analysis suggests that stigma related to STBBI and STBBI testing continues to be a pervasive issue in Métis communities. Participants described how having community members modelling getting tested, and hosting DBST at Métis community events may reduce the stigma associated with HIV/STBBI testing and improve awareness of HIV/STBBI in Métis communities. Similarly, preliminary research in First Nations communities has suggested that a community-level DBS intervention may improve levels of STBBI knowledge and awareness [22]. Additionally, in line with our findings, stigma has been identified in the literature as a barrier to STBBI testing for a variety of populations [12, 39].

### Event-based testing

Participants felt that the ability to host DBST in a Métis community event setting was key to reducing stigma and increasing awareness of STBBI, testing, and treatment in Métis communities which would lead to increased access to culturally safe services for Métis people. The portability of DBST allows for it to be easily offered in community settings such as community events. An additional asset of DBST is that in an event context, many individuals can be tested at a single point in time [40, 41]. Event-based STBBI testing has been used in a variety of contexts and sexual health interventions have been included in some Indigenous community events including at powwows [22, 42]. However, little literature has evaluated DBST in event settings [43].

Additionally, hosting DBST within an event-based setting allows for health role models (i.e. popular opinion leaders) to be present, which may lead to increases in testing service usage. Having popular opinion leaders involved in STBBI interventions is a strategy that has been used with a variety of populations to address stigma associated with STBBIs [44, 45], although no literature specific to Métis communities was identified.

### Métis-specific services and cultural safety

DBST recipients described that having Métis community members trained to provide DBST contributed to their positive experiences with DBST and indicated that it may increase the accessibility of STBBI testing services for Métis community members. Similarly, DBST providers highlighted the importance of DBST being provided by Métis people for Métis people, based on feedback they received from DBST recipients, as they felt this contributed to an improved sense of comfort (via reduced apprehension and fears around experiencing judgement) among test recipients. Participants saw DBST as an opportunity to provide Métis-specific testing services. A lack of Métis-specific health services has been identified as an issue contributing to gaps in health outcomes by many researchers and community organizations, as a lack of culturally-safe services has been shown to impact the way Métis people access health services [5, 17, 19, 46–49]. In the context of Indigenous HCV interventions, Indigenous-led, culturally safe and responsive care has been identified as being more effective than mainstream care, but is constrained by a lack of resources and Indigenous or allied health care practitioners [22, 50]. Due to its portability and since non-healthcare practitioners can be trained to offer DBST, this testing modality allows for increased opportunities for Métis-specific services. Thus, DBST provides an opportunity to address this gap by increasing the accessibility of Métis-specific HIV and STBBI services. Additionally, this model may also increase cultural safety for other Indigenous Peoples if implemented within their communities.

### Limitations

This study is subject to several limitations. Due to the pilot nature of the DBST intervention, our study had a modest sample size, and participants self-selected into the survey and gathering circles. Thus, our conclusions are tentative. It should be noted sampling bias also was inherent to the intervention at several stages, including self-selection of those who attended the community event, as well as for those who chose to test for STBBI with DBST. Still, results suggest that even among this self-selected group, for a significant percentage (approximately 50%) DBST was an opportunity to test for STBBI for the first time. Additionally, given the sensitive nature of this research topic, it is likely only those who were comfortable with speaking about HIV and other STBBI participated in the survey and gathering circles. Similarly, the group-based nature of the gathering circles may have prevented some participants from sharing openly. Additionally, most participants in the community events – and therefore the DBST pilots and this research – were Métis women. Our small sample did not allow for differences in sex or gender to be explored across the sample. Further research should include Métis men, and lesbian, gay, bisexual, transgender, queer and two-spirit identifying Métis to better understand their perspectives on DBST.

## Conclusions

Overall, the findings of this study demonstrate the potential of DBST to be an acceptable, accessible, and culturally safe approach to STBBI testing which can address the unique contexts of Métis communities. DBST has the potential to reduce barriers to STBBI testing for Métis communities. The portability of DBST may increase visibility of STBBI testing and contribute to decreasing stigma related to STBBI testing. Additionally, since DBST can be provided by trained, non-clinical personnel outside a clinical setting, it allows for STBBI testing to be provided in a Métis-specific and culturally safe settings, and can reduce additional barriers to testing for Métis people such as geography, transportation, time and cost associated with regular phlebotomy tests. Additionally, although further research is required, DBST for STBBI may be a promising strategy for increasing access to acceptable, culturally safe services for Indigenous Peoples more broadly.

## Supplementary Information

**Additional file 1: ** The COREQ Checklist was used to guide the reporting of this study.

## Data Availability

The datasets generated and/or analysed during the current study are not publicly available due to good ethical practice for doing research with Indigenous communities following the principles outlined in the Tri-Council Policy Statement: Chapter 9: Research Involving the First Nations, Inuit and Métis Peoples of Canada, but limited additional resources are available from the corresponding author on reasonable request.

## Abbreviations

AIDS: Acquired immunodeficiency syndrome
AHS: Alberta Health Services
COREQ: Consolidated criteria for reporting qualitative research
DBST: Dried blood spot testing
HBV: Hepatitis B Virus
HCV: Hepatitis C Virus
HIV: Human Immunodeficiency Virus
NLHRS: National Laboratory for HIV Reference Services
Shining Mountains: Shining Mountains Living Community Services
STBBI: Sexually transmitted and blood borne infections
