# Supplementary material for: Assessing the acceptability of dried blood spot testing for HIV and STBBI among Métis people in a community driven pilot project in Alberta, Canada
Source: BMC Health Serv Res. 2022 Dec 8;22:1496. doi: 10.1186/s12913-022-08763-z (PMC9733141; doi:10.1186/s12913-022-08763-z)
Supplement: Supplementary file 1 — Additional file 1: The COREQ Checklist was used to guide the reporting of this study. [file 12913_2022_8763_MOESM1_ESM.pdf]

Additional file 1

Assessing the Acceptability of Dried Blood Spot Testing for HIV and STBBI among Métis Peoples: COREQ (Consolidated criteria for Reporting Qualitative research) Checklist

Developed from: Tong A, Sainsbury P, Craig J. Consolidated criteria for reporting qualitative research (COREQ): a 32-item checklist

Topic	Item No.	Guide Questions/Description	Reported on Page No.
**Domain 1: Research team and reflexivity**			
Personal characteristics			
Interviewer/facilitator	1	Which author/s conducted the interview or focus group?	8
Credentials	2	What were the researcher's credentials? E.g. PhD, MD	7-8
Occupation	3	What was their occupation at the time of the study?	7-8
Gender	4	Was the researcher male or female?	7-8
Experience and training	5	What experience or training did the researcher have?	8
Relationship with participants			
Relationship established	6	Was a relationship established prior to study commencement?	8
Participant knowledge of the interviewer	7	What did the participants know about the researcher? e.g. personal goals, reasons for doing the research	8
Interviewer characteristics	8	What characteristics were reported about the inter viewer/facilitator? e.g. Bias, assumptions, reasons and interests in the research topic	8
**Domain 2: Study design**			
Theoretical framework			
Methodological orientation and Theory	9	What methodological orientation was stated to underpin the study? e.g. grounded theory, discourse analysis, ethnography, phenomenology, content analysis	7
Participant selection			
Sampling	10	How were participants selected? e.g. purposive, convenience, consecutive, snowball	8
Method of approach	11	How were participants approached? e.g. face-to-face, telephone, mail, email	8
Sample size	12	How many participants were in the study?	9-10
Non-participation	13	How many people refused to participate or dropped out? Reasons?	10
Setting			
Setting of data collection	14	Where was the data collected? e.g. home, clinic, workplace	8
Presence of non-participants	15	Was anyone else present besides the participants and researchers?	8
Description of sample	16	What are the important characteristics of the sample? e.g. demographic data, date	9-10
Data collection			
Interview guide	17	Were questions, prompts, guides provided by the authors? Was it pilot tested?	8
Repeat interviews	18	Were repeat inter views carried out? If yes, how many?	n/a
Audio/visual recording	19	Did the research use audio or visual recording to collect the data?	9
Field notes	20	Were field notes made during and/or after the inter view or focus group?	n/a
Duration	21	What was the duration of the inter views or focus group?	9
Data saturation	22	Was data saturation discussed?	n/a
Transcripts returned	23	Were transcripts returned to participants for comment and/or	n/a

Topic	Item No.	Guide Questions/Description	Reported on Page No.
		correction?	
**Domain 3: analysis and findings**			
Data analysis			
Number of data coders	24	How many data coders coded the data?	9
Description of the coding tree	25	Did authors provide a description of the coding tree?	n/a
Derivation of themes	26	Were themes identified in advance or derived from the data?	9
Software	27	What software, if applicable, was used to manage the data?	9
Participant checking	28	Did participants provide feedback on the findings?	n/a
Reporting			
Quotations presented	29	Were participant quotations presented to illustrate the themes/findings? Was each quotation identified? e.g. participant number	12-15
Data and findings consistent	30	Was there consistency between the data presented and the findings?	17-20
Clarity of major themes	31	Were major themes clearly presented in the findings?	17-20
Clarity of minor themes	32	Is there a description of diverse cases or discussion of minor themes?	17-20

Developed from: Tong A, Sainsbury P, Craig J. Consolidated criteria for reporting qualitative research (COREQ): a 32-item checklist for interviews and focus groups. *International Journal for Quality in Health Care*. 2007. Volume 19, Number 6: pp. 349 – 357

Additional File

Additional File 1

PDF (.pdf)

Description: The COREQ Checklist was used to guide the reporting of this study.
